# Ultrahigh refractive index sensing performance of plasmonic quadrupole resonances in gold nanoparticles

**DOI:** 10.1186/1556-276X-9-187

**Published:** 2014-04-22

**Authors:** Zehui Yong, Dang Yuan Lei, Chi Hang Lam, Yu Wang

**Affiliations:** 1 Department of Applied Physics and Materials Research Center, The Hong Kong Polytechnic University, Hong Kong, SAR, China

**Keywords:** Localized surface plasmon resonance, Gold nanoparticles, Refractive index sensing

## Abstract

The refractive index sensing properties of plasmonic resonances in gold nanoparticles (nanorods and nanobipyramids) are investigated through numerical simulations. We find that the quadruple resonance in both nanoparticles shows much higher sensing figure of merit (FOM) than its dipolar counterpart, which is attributed mainly to the reduction in resonance linewidth. More importantly, our results predict that at the same sensing wavelength, the sensing FOM of the quadrupole mode can be significantly boosted from 3.9 for gold nanorods to 7.4 for gold nanobipyramids due to the geometry-dependent resonance linewidth, revealing a useful strategy for optimizing the sensing performance of metal nanoparticles.

## Background

Localized surface plasmon resonances (LSPRs) are optical phenomena that occur in metallic nanoparticles in which collective charge motions confined at metal-dielectric interfaces can be driven into a resonant state by an incident light at a particular wavelength and polarization state. Their unique properties such as increased absorption/scattering cross section and enhanced local electromagnetic fields make them extremely versatile in a wide range of applications in nanophotonics [1] and biochemical sensing [2,3]. For example, one typical application of LSPRs is the refractive index (RI) sensing, which utilizes the peak shift in the extinction spectrum of metal nanoparticles due to the RI change of the surrounding environment. A widely used figure of merit (FOM) parameter that characterizes the LSPR sensing capability is given as [3,4].

(1) $$\mathrm{FOM}=\frac{1}{\Delta \lambda }\frac{d\lambda_{sp}}{dn},$$

where *λ*_sp_ and *n* are the resonance wavelength and the surrounding RI, respectively; d*λ*_sp_/d*n* and Δ*λ* are the RI sensing sensitivity and the resonance linewidth, respectively.

It is well known that the resonant feature of LSPR is highly sensitive to the size, material, and the shape of nanoparticles [3,5]. This property has stimulated a great deal of efforts in searching for optimal nanoparticle geometries for LSPR sensing. In general, it is believed that irregular shapes perform better than conventional nanospheres, particularly for those containing sharp tips [2,6]. For example, it has been shown that the sensing FOM of gold nanobipyramids (1.7 ~ 4.6) [7,8] and nanostars (3.8 ~ 10.7) [6,9] is much larger than that of ordinary shapes such as nanospheres (0.6 ~ 1.5) and nanorods (1.3 ~ 2.1) [3,7]. However, practical applications are facing a trade-off between synthesis difficulties and the sensing performance, since synthesis of complex morphologies often needs delicate controls over the reaction conditions and usually results in a low reproducibility [10-12]. Other approaches for better RI sensing include introducing nanocavities [13,14], or fabricating particularly designed nanoparticles [15,16], where even more complicated fabrication efforts are required. Therefore, it is beneficial to search for new routes to improve the sensing performance of LSPRs.

In the past, LSPR sensing studies have mostly focused on the use of the fundamental dipole mode, while higher order resonances have received relatively little attention due to the fact that chemical synthesis tends to produce small-sized (compared to wavelength) nanoparticles. Some pioneering studies on exploration of higher order resonances include dipole-quadrupole interactions [17], Fano resonance [18], and also dipole-propagating mode coupling [19,20]. In this letter, we show, through comprehensive numerical studies, that higher order resonances in gold nanoparticles (particularly the quadruple mode in gold nanobipyramids) are significantly superior to dipolar resonances in LSPR sensing, thus avoiding assiduous tailoring of nanoparticle geometries.

## Methods

The optical properties of gold nanoparticles are solved numerically in the frequency domain using the scattered field formulation. Field analysis was performed using a commercially available finite-element-method package (COMSOL Multiphysics 4.3a). The simulation method has been well documented in [21-23]. The extinction cross section is simply defined as the sum of absorption and scattering cross sections of the nanoparticles. More specifically, the dielectric function of gold used in the simulations is extracted by interpolation of Johnson and Christy's results [24], and the nanoparticles are placed in a homogeneous medium resembling water, whose RI can be changed from 1.33 to 1.37 for comparison.

## Results and discussion

### Multipolar plasmonic modes in gold nanorods

**Figure 1:**
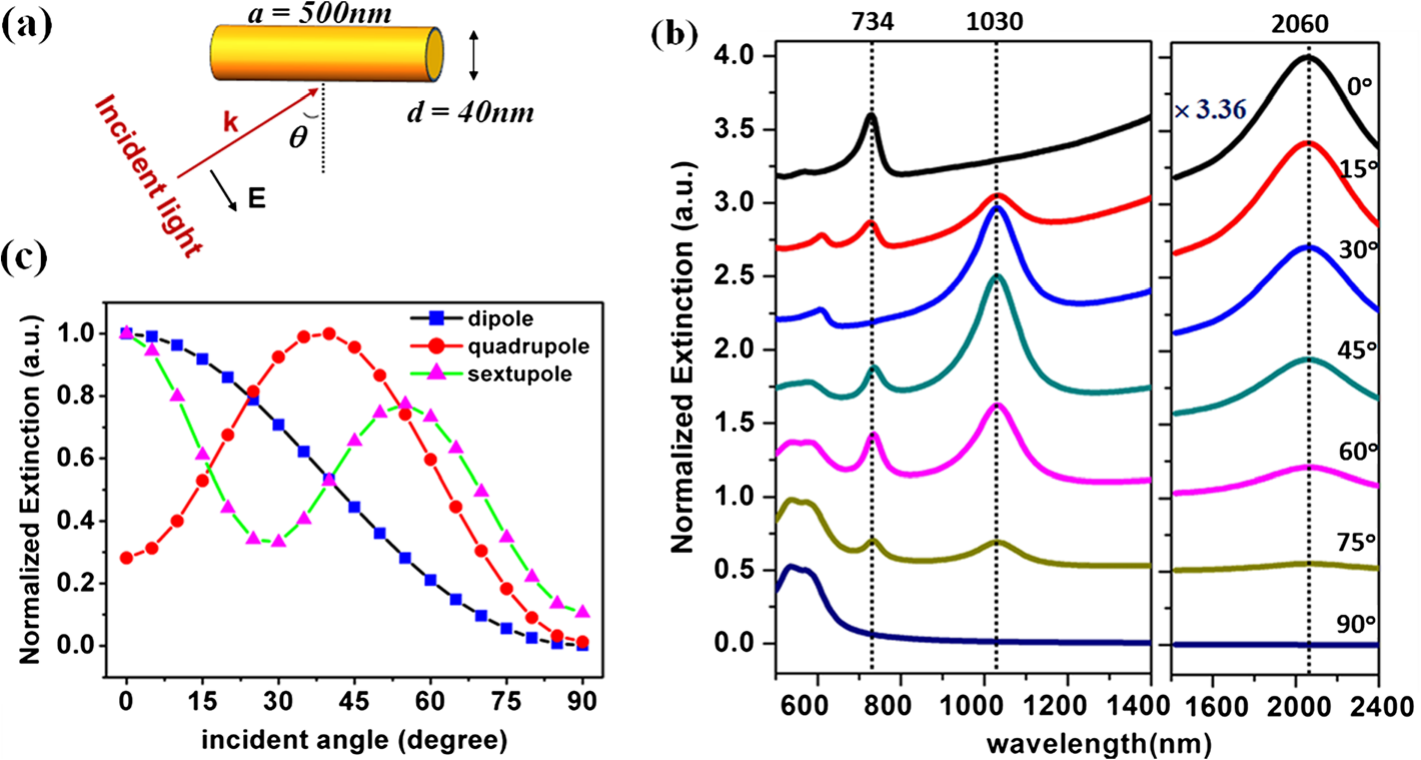
**Extinction characteristics of a gold nanorod in water (*n*** **= 1.33). (a)** The configuration of the numerical modeling. **(b)** Simulated extinction spectra of the gold nanorod for different incident angles *θ*; the extinction value in the left panel is normalized to the quadrupole peak for *θ* = 45°, and in the right panel to the dipole peak for *θ* = 0° (with a scale 3.36 times larger than the left panel). Curves are plotted with offset for clarity. **(c)** Angle-dependent peak extinction for the dipole, quadrupole, and sextupole resonance modes, normalized to the maximum values of each mode.

Excitations of plasmonic higher order modes such as quadrupole and sextupole resonances in metallic nanoparticles require a particular incident angle and polarization state. Figure 1a shows an angle-dependent excitation of a gold nanorod (length 500 nm, diameter 40 nm) in water (*n* = 1.33) by a TM-polarized plane wave.

**Figure 2:**
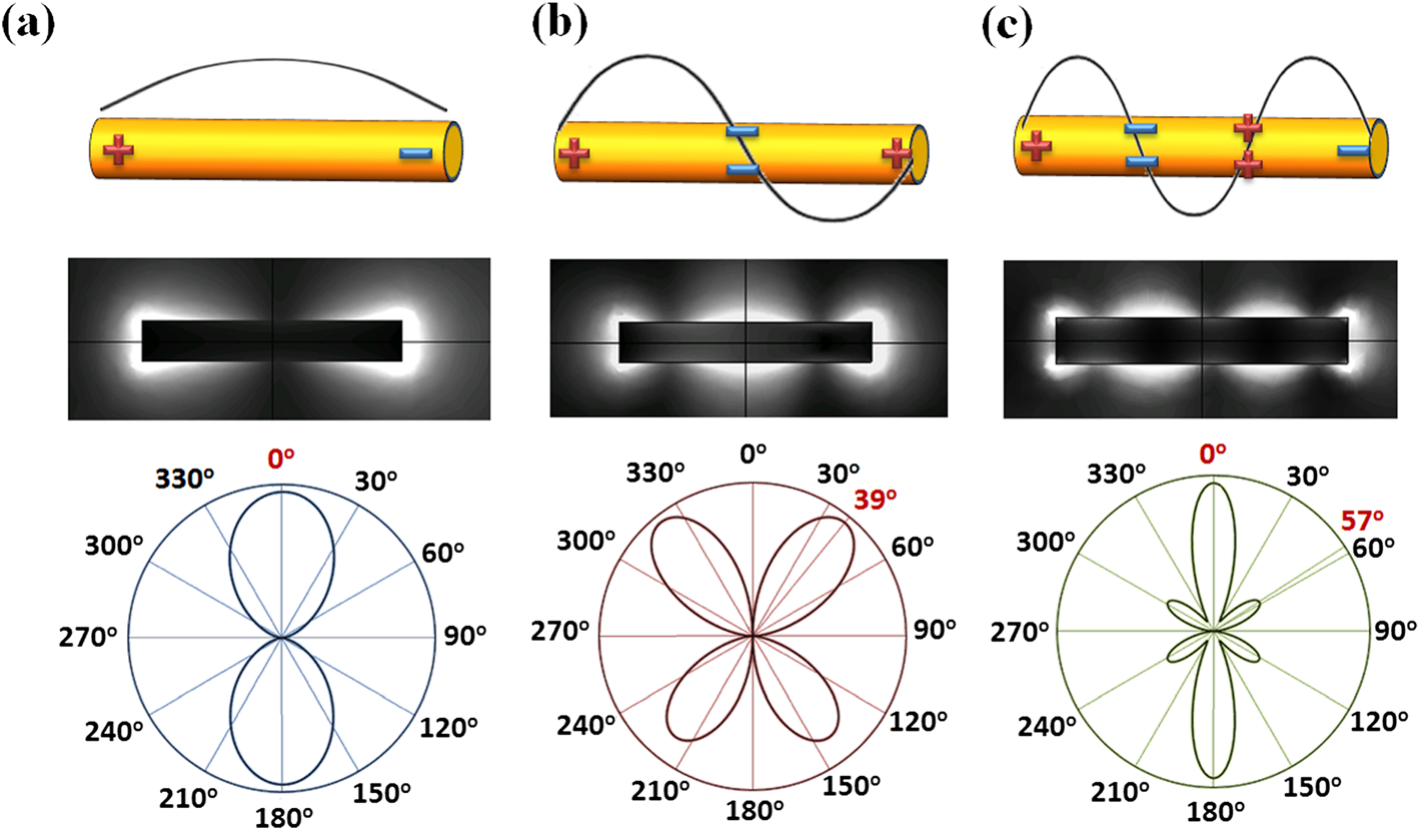
**LSPR schematics.** Schematic charge distribution, electric near-field amplitude distribution, and far-field scattering radiation pattern of a gold nanorod upon excitations of **(a)** its dipole mode (2,060 nm), **(b)** quadrupole mode (1,030 nm), and **(c)** sextupole mode (734 nm). Red numbers in the scattering patterns indicate the angles with maximal scattering power.

Figure 1b renders the extinction spectra of a gold nanorod at different excitation angles, which show three distinct extinction peaks, namely a dipole resonance at 2,060 nm, a quadrupole resonance at 1,030 nm, and a sextupole resonance at 734 nm, respectively. The mode nature of these three extinction resonances is unambiguously confirmed respectively by their near-field distribution (electric field amplitude) and far-field radiation patterns, as shown in Figure 2. The extinction spectra shown in Figure 1b also reveal that each resonance has an optimal excitation angle at which the extinction cross section is a maximum. The normalized extinction intensity for each resonance is plotted as a function of the incident angle as shown in Figure 1c. As expected, the dipole resonance is efficiently excited when the incident polarization is parallel to the nanorod axis. Interestingly, the quadrupole mode responds most strongly to an incident angle at 40°, while the sextupole mode shows double maxima at excitation angles of 0° and 55°. In fact, these optimal angles correspond, respectively, to the maximum near-field amplitude and far-field radiation power for each resonance presented in Figure 2. Other higher order modes and the traverse mode can also be observed at the short wavelength limit, which is out of the scope of this paper.

### Sensitivities of quadrupole resonances

In the following, we will investigate the extinction response of four types of gold nanorods and compare their RI sensing performance. The structures under study are as follows: type A, gold nanorod with *a* = 200 nm and *d* = 80 nm; type B, gold nanorod with *a* = 500 nm and *d* = 80 nm; type C, gold nanobipyramid with *a* = 200 nm and *d* = 100 nm; and type D, gold nanobipyramid with *a* = 200 nm and *d* = 42.5 nm. The dimensions of these nanorods are chosen such that the dipole resonance wavelength of types A and C and the quadrupole resonance wavelength of types B and D are all around 1,050 nm in order to compare their RI sensing sensitivities at the same wavelength. The geometry of nanobipyramids is selected because of its high FOM as reported previously [7,8]. To avoid numerical errors caused by the sharp tips and to be more realistic to the experimental samples, the edges of the two tips in nanobipyramids are blunted with a frustum shape.

**Figure 3:**
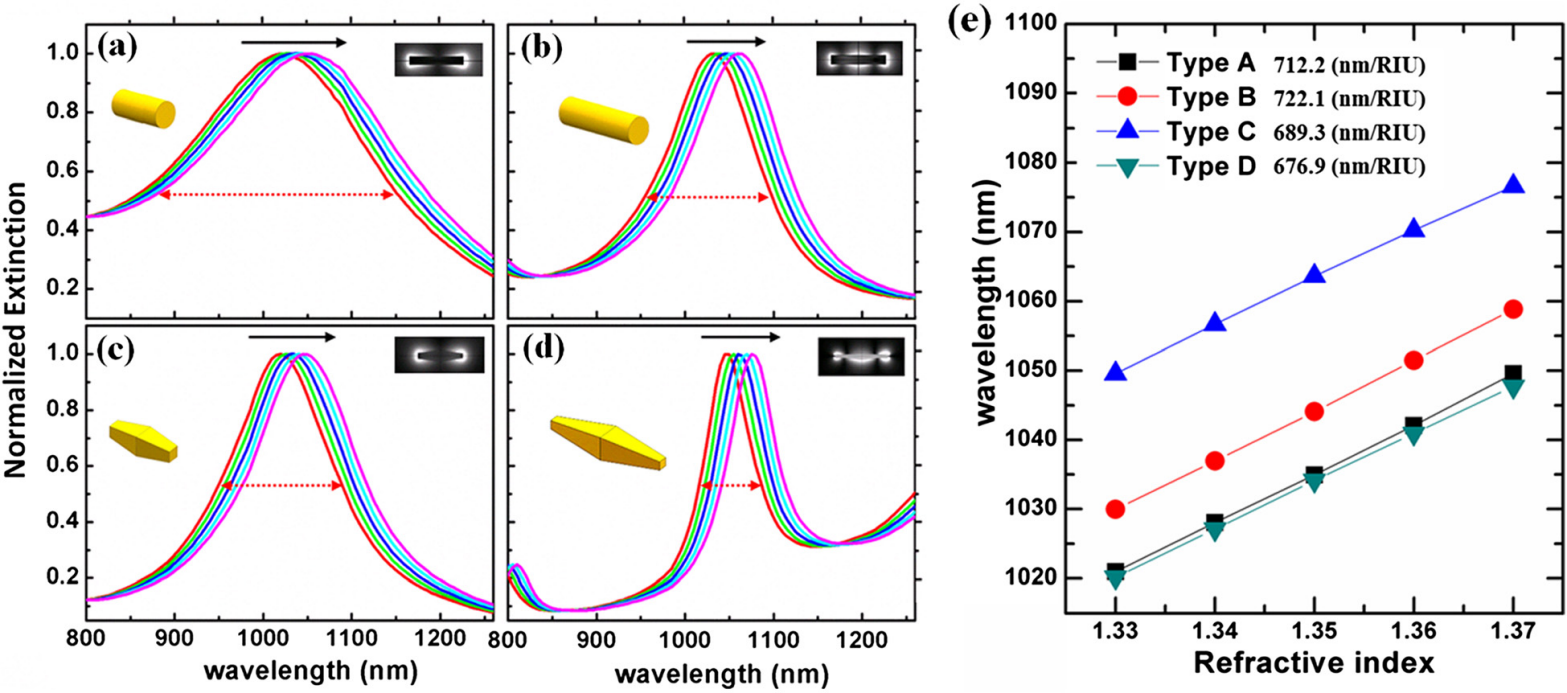
**RI-dependent extinction spectra.** Near the **(a, c)** dipole resonance mode of nanorods of types A and C and **(b, d)** quadruple resonance mode of nanorods of types B and D, respectively, with all the structures in a surrounding medium of RI varying from 1.33 to 1.37. The black arrows represent the shifting direction of the resonance peak from the case RI = 1.33 to RI = 1.37. The red double arrows denote the linewidth of each peak. Insets are schematics of nanoparticle geometries and their electric near-field amplitude distributions at the corresponding LSPR wavelengths. **(e)** Peak wavelengths *λ*_sp_ as a function of the surrounding RI for different LSPR modes/shapes corresponding to **(a)** to **(d)**. The RI sensitivities d*λ*_sp_/d*n* of the four curves are 712.2, 722.1, 689.3, and 676.9, in the unit of nm/RIU, respectively.

By changing the RI of the surrounding medium from 1.33 to 1.37 (supposing a fixed incident angle = 60°), the extinction peak (*λ*_sp_) of each nanorod gradually redshifts towards a longer wavelength, as shown in Figure 3a,b,c,d. These results are summarized in Figure 3e in which the extinction peak for each nanorod is plotted as a function of the refractive index. It can be observed from Figure 3e that the slopes of the four curves - which directly represent the RI sensitivity d*λ*_sp_/d*n* - are not substantially different from each other, in an obvious contradiction to previous reports [3,6-8]. This observation is due to the fact that the RI sensitivity of LSPRs is actually wavelength dependent, which means that the RI sensitivity will not depend much on the mode resonance of choice or the structure geometry once the sensing wavelength is fixed (consistent with previous theoretical results by quasi-static approximation [25,26]). This also points out that it might be inappropriate to compare directly the RI sensitivities of LSPRs of different nanostructures at different wavelengths [3,6-11,13-17]. We also refer to the article [27], where the authors have argued that any single mode sensing of RIs such as LSPR sensing cannot surpass an upper limit of *λ*/*n*, where *λ* is the sensing wavelength and *n* is the surrounding RI - which means an upper limit of 1,050 nm/1.33 = 789.5 nanometer per RI unit (nm/RIU) for our case. Therefore, further efforts to improve the RI sensitivity of LSPRs are probably not practical. Accordingly, some results above this theoretical limit obtained from some particular nanostructures such as nanostars [6] may be attributed to a collective excitation of multiple LSPR modes (though in single nanoparticles), or other chemically induced effects. Our calculations also show that the RI sensitivity is independent of *θ* (results not shown here). Therefore, the conclusion from Figure 3e must hold true for any incident angles and also for random orientation of nanoparticles.

### Linewidths of quadrupole resonances

As mentioned earlier, the resonance linewidth is the other important factor in determining the overall RI sensing performance of LSPRs [28]. Opposite to the RI sensitivity, the resonance linewidth of LSPRs largely depends on the incident angle, as demonstrated in Figure 1b. In addition, for LSPR sensing measurements with typical experimental setups [28], the characterization results are in fact collective effects arising from the total response of a mass of randomly oriented nanoparticles. Therefore, it is necessary to average the linewidth of the simulated extinction spectra at different excitation angles for each structure.

**Figure 4:**
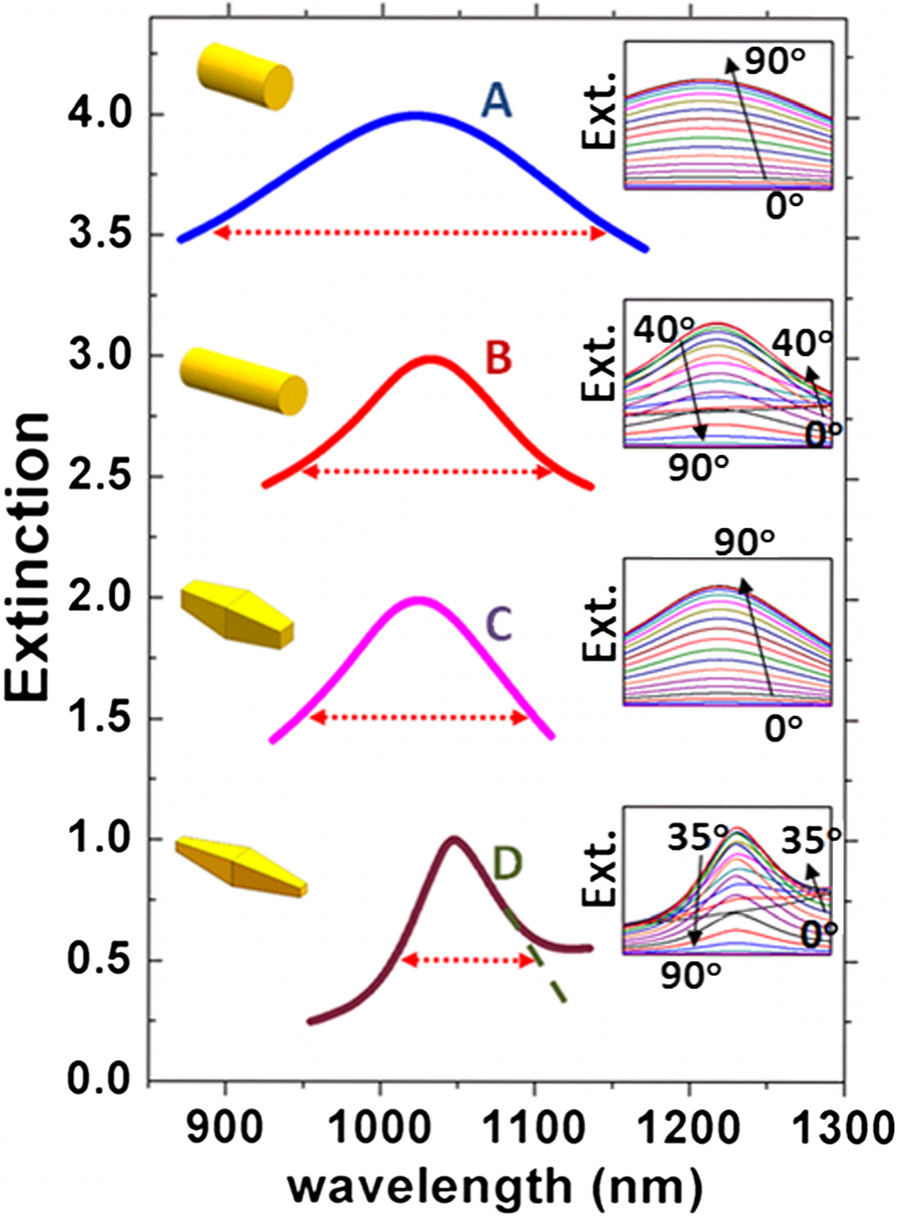
**Incident angle-averaged extinction spectra.** Normalized incident angle-averaged extinction spectra for nanorods of types A, B, C, and D in the wavelength of interest, with surrounding medium of RI = 1.33. The red double arrows denote the fullwidth at half maximum linewidth of each peak. For the D curve, the extrapolation line is also shown. The curves are plotted in offset for clarity, with insets showing the schematics of the nanorods (left) and their angle-dependent extinction spectra (right).

The incident angle-dependent extinction spectra for the four types of Au nanorods are presented in the insets of Figure 4, and the curves in each inset are summed and averaged for calculating the average resonance linewidth, as shown in the main panel of Figure 4. It can be seen that the averaged extinction spectra for nanorods of type A, B, and C are all symmetric with a well-defined resonance linewidth (i.e., full width at half maximum), while the spectrum of type D nanorod exhibits a largely asymmetric profile and needs an extrapolation to extract the resonance linewidth. The resulting resonance linewidths for the four nanorods are 278.6, 186.8, 154.1, and 91.7 nm, respectively. An obvious observation is that the resonance linewidth reduces from dipole modes (types A and C) to quadrupole modes (types B and D) and also from regular nanorod shapes to irregular nanobipyramid shapes. Note that the nanobipyramid of type D has the narrowest resonance linewidth, which is due mainly to the coupling between its quadrupole and dipole resonances that are close to each other in wavelength. This possesses similar characteristics to Fano resonances in which the electromagnetic coupling between a dark mode with narrow resonance linewidth and a bright mode with a broad resonance linewidth creates a sharp Fano dip in the spectrum, which can be used to enhance the sensing FOM [18]. A similar coupling effect has also been observed for propagating surface plasmons and waveguide modes in one-dimensional periodic metal grooves [29]. We have to point out that the linewidth reduction observed here may be the main contribution to the reported FOM enhancements [6-9].

### FOM of quadrupole resonances

Finally, we calculated the overall sensing FOM in terms of the RI sensing sensitivity and the extracted resonance linewidth, with results summarized in Table 1 in which some data from literature are also added for reference. For plasmonic dipole modes, the FOM values derived from our numerical methods are partially consistent with previous experimental results. A slightly larger FOM observed for the nanorod dipole mode in our studies may be due to the sharp edges of the rod defined in our simulation model. For quadrupole modes, we estimated an FOM of 3.9 for the nanorod of type B and 7.4 for the nanobipyramid of type D, both much larger than the FOM values [3,6-9] reported for dipole modes in the both structures, suggesting the great promise of using quadruple resonances in single-particle RI sensing.

**Table 1:** Comparison of RI sensing performance for different nanoparticles

**Type**	**Mode**	**Size**^**a **^**(nm)**	***λ***_**sp **_**(nm)**	**d** ***λ***_**sp**_**/d** ***n***^**b**^	**Δ** ***λ *** **(nm)**	**FOM**
Nanorod (A)	D	200/80	1,020	712.2	278.6	2.6
Nanorod (B)	Q	500/80	1,030	722.1	186.8	3.9
Nanobipyramid (C)	D	200/100	1,020	689.3	154.1	4.5
Nanobipyramid (D)	Q	200/42.5	1,045	676.9	91.7	7.4
Nanorod [7]	D	55/16	728	224		2.1
Nanorod [11]	D	50/15	730	170	125	1.3
Nanobipyramid [7]	D	189/40	1,098	540		4.5
Nanobipyramid [8]	D	90/30	800	352		4.5

^a^The nanoparticle sizes are expressed in the form of length/diameter. ^b^The unit for RI sensitivity is nanometers per refractive index unit (nm/RIU). D, dipole mode; Q, quadrupole mode.

## Conclusions

In conclusion, we have demonstrated an ultrahigh overall sensing figure of merit by using plasmonic quadrupole resonances in gold nanorods and nanobipyramids. Three important conclusions can be drawn from our detailed numerical studies: (1) The excitation efficiency of LSPRs in nanorods by plane waves exhibits an angle-dependent behavior, which is consistent with their electric near-field enhancements and far-field scattering radiation patterns. (2) The refractive index sensitivity of single-mode LSPR in nanoparticles is independent of the resonance mode of choice and the particle geometry provided that the sensing wavelength is fixed. (3) The improved FOM observed for plasmonic quadrupole resonances in gold nanoparticles in the present work as well as in previous studies is due mainly to the reduction of resonance linewidth. Our results suggest that plasmonic quadrupole modes in gold nanorods are possibly the most promising choice to achieve the best sensing performance and that it is of particular importance to explore multipolar resonances for further sensing studies.

## Abbreviations

FOM: figure of merit; LSPR: localized surface plasmon resonance; nm/RIU: nanometer per refractive index unit; RI: refractive index.

## Competing interests

The authors declare that they have no competing interests.

## Authors' contributions

ZY carried out the calculation and data analysis and drafted the manuscript. DYL conceived the project and co-wrote the manuscript. CHL and YW participated in the discussion and revisions. YW participated in the coordination. All authors read and approved the final manuscript.
